# Cross-Breeding Improvement and Performance Analysis of Dominant Production Traits in Grazing-Type Alfalfa (*Medicago sativa L*.)

**DOI:** 10.1155/2022/1252310

**Published:** 2022-11-10

**Authors:** Yun A, Shangli Shi, Wenlong Gong, Jinqing Zhang, Xiaoyan Zhang, Jing Zhang

**Affiliations:** ^1^College of Grassland Science, Gansu Agricultural University/Key Laboratory of Grassland Ecosystem, Ministry of Education/Sino-U.S. Center for Grassland Ecosystem Sustainability, Lanzhou, Gansu 730070, China; ^2^Lanzhou Institute of Husbandry and Pharmaceutical Sciences of CAAS, Lanzhou, Gansu 730070, China

## Abstract

Rhizome-rooted *Medicago sativa L*. “Qingshui” is an excellent germplasm for establishing grazing and ecological grasslands but inferior in yield, in which both high production and ecological values can be achieved by cross-breeding. We have obtained valuable rhizome-rooted hybrid strains (RSA-01, RSA-02, and RSA-03) by crossing of Qingshui and the high-yielding *Medicago sativa L*. “WL168.” In this study, the Qingshui plants with low production performance were crossed for improvement, and progenies with better production and higher quality than those of Qingshui were selected. The results reveal that the branch number, crude protein (CP) content, and relative feed value (RFV) of RSA-01; the stem thickness, CP content, and ether extract (EE) content of RSA-02; and the plant height, stem thickness, branch number, and dry hay yield of RSA-03 were higher than those of Qingshui. Except for the leaf/stem ratio and plant height of RSA-01, leaf/stem ratio of RSA-02, and plant height of RSA-03, the coefficient of variation (CV) of yield traits of the hybrid strains was lower than those of Qingshui, ranging from 0.1% to 4.28%. In addition to the lignin and acid detergent fiber content of RSA-01 as well as EE content of RSA-02 and RSA-03, the CV of the nutritional traits of the hybrid strains was low, ranging from 0.60% to 3.43%. The tested samples were ranked as follows based on yield performance and nutritional values: WL168 > RSA − 03 > RSA − 01 > RSA − 02 > Qingshui and RSA − 01 > WL168 > RSA − 03 > Qingshui > RSA − 02, respectively. Compared with parental Qingshui, RSA-01, RSA-02, and RSA-03 show better yield performance; meanwhile, RSA-01 and RSA-03 had higher nutritional traits. RSA-01 shows heterosis in branch number, CP content, and RFV; RSA-02 shows heterosis in stem thickness and RSA-03 in plant height, stem thickness, branch number, fresh yield, dry hay yield, and CP content. Notably, the low production performance of Qingshui was improved after crossing it with WL168, substantially resulting in an abundant rhizome-rooted germplasm resource for the establishment of grazing grasslands.

## 1. Introduction

Cultivation and development of forage alfalfa can enhance the productivity of grassland–livestock systems and achieve ecological protection [1]. Alfalfa is the most widely cultivated and utilized leguminous forage worldwide, with a large number of species and varieties [2]. Based on their morphology, alfalfa roots were classified into four categories: tap-rooted, branch-rooted, creeping-rooted, and rhizome-rooted [3]. Rhizome-rooted and creeping-rooted alfalfa, also known as “grazing” alfalfa, are suitable for establishing grazing pastures as they are ecologically beneficial owing to their well-developed rhizomes and creeping root systems, highly resistant to trampling and robust renewable [4–6]. Studies on breeding of grazing alfalfa varieties date back to the early 20th century in the former Soviet Union and Canada [4]. The current global research and use of grazing alfalfa are primarily based on the creeping-rooted varieties, including *Medicago varia* Martyn “Tumu No. 3,” *Medicago sativa L*. “Tumu No. 4,” *Medicago falcata* “Hulunbeier,” and *Medicago varia* Gannong “No. 2,” which are found in China [7], as well as Rambler, Rangelander, and Camporegio from other countries [8]. The rhizome of rhizome-rooted alfalfa has higher renewal and greater expansion capacity, with better ground cover formation than that of creeping-rooted alfalfa. Currently, *Medicago sativa* L. “Qingshui” is the only validated and registered (registration number 412) rhizome-rooted alfalfa species in China, which warrants its exploitation owing to the high-quality grazing forage, slender and stiff stalks, lax plant shape, and horizontal or oblique branches and roots (Table S1 summarizes the characteristics of each variety) [9–12]. Qingshui is an exceptional germplasm resource with relatively high resistance to stress and trampling as well as high renewal capacity but low forage yield [12]. Previous studies on this germplasm included karyotype analysis, enzyme profiling, DNA molecular marker identification, tissue dissection, and leaf metagenesis patterns during shoot development from rootstocks which were examined [12, 13]; however, no study on yield performance after breeding improvement is available in the literature.

Cross-breeding is a common and effective method of alfalfa breeding. Plant varieties with elite traits are selected to form a hybrid group [14]. Subsequently, variants with heterosis are isolated after cross-breeding and then screened for new varieties with unprecedented phenotypic traits. The main objective of cross-breeding in alfalfa is to transfer superior genes into the candidate gene pool and to rapidly create new germplasm with superior performance [15]. Over the years, scientists have bred many excellent varieties through cross-breeding methods. For example, Wang et al. [16] crossed a semisibling progeny of 16 excellent alfalfa strains to screen productive and high-quality progenies, including fast-growing #15 and fast-growing #12.

Grazing alfalfa has prolonged lifespan, high and stable yield, and good quality traits. This study is aimed at improving low production performance of Qingshui varieties by cross-breeding, and WL168 was selected as the parent that provided excellent traits. RSA-01, RSA-02, and RSA-03 as were used the research objects, and the parental Qingshui was used as the control. We measured the yield-related and nutrient-related indices and analyzed their dominant trait and the degree of variation. Subsequently, the progenies with better production performance and quality values than those of Qingshui were selected. The hybrid strains are expected to achieve the goal of high production and ecological values, meanwhile potentially providing an abundant rhizome-rooted germplasm resource for the establishment of forage systems.

## 2. Materials and Methods

### 2.1. Test Varieties

The target variety for improvement was the rhizome-rooted *Medicago sativa* L. “Qingshui,” whereas the parental variety possessing excellent traits was the creeping-rooted *Medicago sativa* L. “WL168.” Using WL168 (male) and Qingshui (female), a single cross combination was performed to obtain RSA-01, RSA-02, and RSA-03. Phenotypic variations of the three hybrid strains were analyzed, and the parental Qingshui was used as the control sample.

The origin and development of the hybrid strains were same as the previous research [17, 18]. Basic traits for each strain are presented in Table S1 [11]. WL168 was provided from Beijing Zhengdao Ecological Technology Co., Ltd., and Qingshui was provided by the College of Grassland Science of Gansu Agricultural University, China.

### 2.2. Growth Conditions and Treatments

Our test was performed in 2021 at the experimental base in Gansu Agricultural University (34°05′N, 105°41′E, and 1525 m altitude), Lanzhou, China, with a temperate semiarid continental climate, an average annual temperature of 9.7°C, an average annual precipitation of 451.6 mm, and an annual evaporation of 1664 mm. The test site has a flat topology with well-aerated yellow loess soil.

Seeds were sown on April 25, 2016, under field environmental conditions. Five experimental materials consisting of three hybrid strains and two parental varieties were grown in three replicates for each group in a randomized complete block design. The plot area was 3 m × 5 m with a row spacing of 30 cm (sowing density, 1 g/m^2^). Timely field managements including irrigation, manual weeding, and pest control were performed.

Yield performance and nutritional value were observed and measured whenever each cultivar either had 10% of flowering plants in 2021 (May 15, July 20, and September 5), using 5-year-old alfalfa grass plots. Each plot was randomly selected; cuts were made at 5.0 cm above the soil; the samples were weighed and then briefly dried at 105°C for 30 min followed by extended drying to a constant weight at 65°C for 18 h. The dried samples were weighed, crushed, passed through a 1 mm sieve, and stored in a refrigerator at 4°C.

### 2.3. Measurement of Improvement Indicators

#### 2.3.1. Yield-Related Indicators

- Plant height (cm): ten plants were randomly selected in each plot, and their absolute height (vertical distance from the ground to the tip) was measured and averaged.
- Stem thickness (mm): ten plants were randomly taken from each plot, and the diameter of the main stem at 5 cm height above the ground was measured using vernier calipers, and then measurements were averaged.
- Leaf/stem ratio: approximately 500 g of fresh sample was taken from each plot, and the stems and leaves were separated and weighed. Samples were dried in an oven at 105°C for 10 min before drying to a constant weight at 65°C. The leaf/stem ratio (mass of leaves/mass of stems) was calculated in triplicates, and then, measurements were averaged.
- Number of branches: ten plants were randomly selected in each plot, and the number of primary branches in each plant was determined.
- Overwintering rate: the number of plants in each sampling plot was recorded before and after winter to determine the number of green surviving plants in the following year, and the resulting values were used to calculate the overwintering rate of alfalfa using the formula below, as previously described [16].

(1) $$\begin{array}{c} \text{Overwintering rate}=\frac{N\;\left(\text{Number of surviving plants before overwintering}\right)}{N_{1}\;\left(\text{Number of surviving plants after overwintering}\right)}\times 100\%. \end{array}$$

(vi) Fresh yield and dry hay yield (t/hm^2^): for each crop mowing and yield measurement, a 2 m long section was randomly sampled in each plot to obtain a 5 cm stubble, which was weighed immediately after mowing to measure its fresh yield. The dry hay yield was calculated in triplicates according to the fresh/dry ratio, and the average values were recorded.
(vii) Fresh/dry ratio: for each mowing and yield measurement, 500 g of samples was randomly taken from each plot and cut into 4 cm sections. Samples in three replicates were then mixed well, and approximately 1,000 g of these samples was weighed and recorded as fresh weight. After drying and obtaining the dry weight, the fresh/dry ratio was calculated

#### 2.3.2. Quality-Related Indicators

Crude protein (CP), ether extract (EE), crude ash (ash), and lignin (ADL) contents were measured as described by Machado et al. [19], and neutral detergent fiber (NDF) and acid detergent fiber (ADF) contents were measured as described by Tucak [20].

The relative feed value (RFV) was determined using the following equations, as developed by Tucak [20]. Digestible dry matter (DDM) and dry matter intake (DMI) were estimated, following which RFV was calculated.

(2) $$\begin{array}{c} \text{DDM }\left(\%\right)=88.9-\left(0.779\times \%\text{ADF}\right), \end{array}$$

(3) $$\begin{array}{c} \text{DMI }\left(\%\right)=\frac{120}{\%\text{NDF}}, \end{array}$$

(4) $$\begin{array}{c} \text{RFV}=\text{DDM}\times \text{DMI}\times 0.775. \end{array}$$

### 2.4. Data Processing and Analysis

Data from three biological replicates were statistically analyzed using the SPSS 20.0 software, and all improvement indicator values were calculated in Excel 2010 using the formula described in Equations (5) and (6) below. GraphPad Prism 8 was used for mapping, and data were presented mean ± standard error in bar graphs. Statistical analyses were performed by one-way analysis of variance and Duncan's multiple range test at a significance level of *P* < 0.05.

- Calculation of coefficient of variation (CV):

(5) $$\begin{array}{c} S=\sqrt{\frac{1}{N}\overset{N}{\underset{i=1}{\sum }}{\left(Xi-X\right)}^{2}}, \end{array}$$

(6) $$\begin{array}{c} \text{CV}=\frac{\text{S}}{\bar{X}}, \end{array}$$

where *X* represents the specific value of the measured index, $\bar{X}$ represents the average value, *N* represents the number of replicates, and *Xi* represents the *i*-th data.

(ii) Comprehensive evaluation of gray correlation degree [21]:

Standardization of the average value of each index was calculated according to Equation (7). The comprehensive gray correlation degree was calculated according to

(7) $$\begin{array}{c} y_{i}=\frac{x_{i}}{1/n\sum_{i-1}^{n}x_{i}}, \end{array}$$

(8) $$\begin{array}{c} \zeta_{\left(k\right)}=\frac{\min_{i}\min_{k}△i\left(k\right)+\rho \max_{i}\max_{k}△i\left(k\right)}{△i\left(k\right)+\rho \max_{i}\max_{k}△i\left(k\right)}, \end{array}$$

(9) $$\begin{array}{c} r_{i}=\frac{1}{n}\overset{n}{\underset{k=1}{\sum }}\zeta_{i}\left(k\right)\;\left(n=1,2,3,\cdots \right), \end{array}$$

(10) $$\begin{array}{c} w_{i}=\frac{r_{i}}{\sum r_{i}}, \end{array}$$

(11) $$\begin{array}{c} D=\overset{n}{\underset{k=1}{\sum }}w_{i}*\zeta_{\text{i}}\left(k\right), \end{array}$$

where *ζ*_(*k*)_ is the correlation coefficient, △*i*(*k*) denotes the absolute difference between the ideal sequence and test sequence, and *ρ* is the resolution coefficient. When considering a *D* value of 0.5 as the weighted correlation degree, *w*_*i*_ denotes the index weight value, *n* denotes the number of each trait index in the tested sample, and *k* denotes the trait, whereas *i* represents the variety number.

## 3. Results

### 3.1. Analysis of Yield Performance of Hybrid Strains

**Figure 1:**
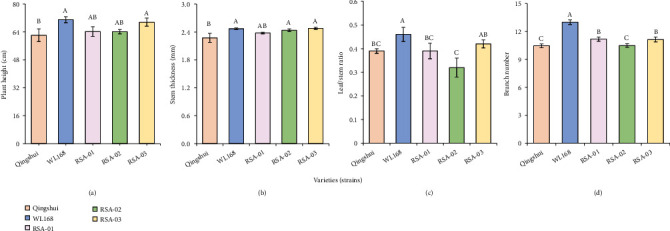
Estimated values of growth parameters in hybrid strains: (a) plant height, (b) stem thickness, (c) leaf/stem ratio, and (d) branch number. Ten plants were randomly selected in each plot, and their growth parameters were measured, and then, the mean value of each parameter was calculated. Different capital letters indicate significant differences among varieties at *P* < 0.05.

To compare the growth parameters of the tested varieties (strains), the plant height, stem thickness, leaf/stem ratio, and branch number of the hybrid strains were measured (Figure 1). The plant heights of RSA-01 (64.22 cm) and RSA-02 (64.14 cm) differ insignificantly from that of Qingshui (62.12 cm), whereas the plant height of RSA-03 (69.60 cm) was significantly higher than that of Qingshui by 12.04% (*P* < 0.05). The stem thickness of RSA-01 (2.38 mm) differs insignificantly from that of Qingshui, whereas the stem thicknesses of RSA-02 (2.44 cm) and RSA-03 (2.48 mm) were significantly higher than that of Qingshui (2.27 mm) by 7.49% and 9.25%, respectively. The leaf/stem ratios of RSA-01 (0.39) and RSA-03 (0.42) differ insignificantly from that of Qingshui (0.39), whereas the leaf/stem ratio of RSA-02 (0.32) was significantly lower than that of Qingshui by 17.95%. The branches' numbers of RSA-01 (11.18) and RSA-03 (11.14) were significantly higher than that of Qingshui (10.46) by 6.88% and 6.50%, respectively, whereas the branch number of RSA-02 (10.50) did not differ significantly from that of Qingshui. The mean plant height (65.99 cm), mean stem thickness (2.60 cm), and mean branch number (10.94) of the three hybrid strains were higher than those of Qingshui by 5.86%, 14.54%, and 4.59%, respectively.

**Figure 2:**
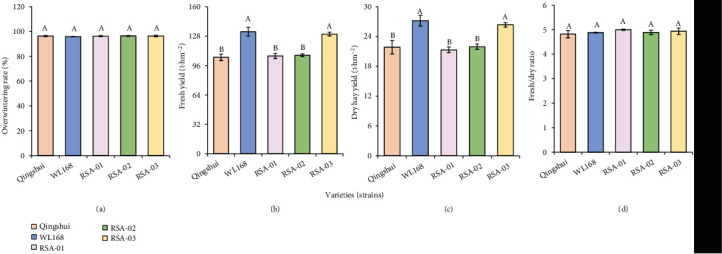
Estimated values of yield traits in hybrid strains: (a) overwintering rate, (b) fresh yield, (c) dry hay yield, and (d) fresh/dry ratio. A 2 m long section was randomly sampled in each plot to obtain a 5 cm stubble; their yield traits were measured, and then, the mean value of each parameter was calculated. Different capital letters indicate significant differences among varieties at *P* < 0.05.

To compare the yield traits of the tested varieties (strains), overwintering rate, fresh yield, dry hay yield, and fresh/dry ratio of the hybrid strains were measured (Figure 2). The overwintering rate (96.26%-96.44%) and fresh/dry ratio (4.89-5.00) of the three hybrid strains differ insignificantly from those of Qingshui (*P* ≥ 0.05). Similarly, fresh yields of RSA-01 and RSA-02 differ insignificantly from that of Qingshui (*P* ≥ 0.05). In contrast, the fresh yield of RSA-03 (130.12 t·hm^−2^) was significantly higher than that of Qingshui (104.93 t·hm^−2^) by 24.01% (*P* < 0.05). The differences in dry hay yield among RSA-01 (21.29 t·hm^−2^), RSA-02 (21.94 t·hm^−2^), and Qingshui were insignificant, whereas the dry hay yield of RSA-03 (26.36 t·hm^−2^) was significantly higher than that of Qingshui (21.85 t·hm^−2^) by 20.64%. The mean fresh yield (114.58 t·hm^−2^) and mean dry hay yield (23.20 t·hm^−2^) of the three hybrid strains were higher than those of Qingshui by 9.20% and 6.16%, respectively.

To measure the degree of variation in yield performance indicators among the hybrid strains, the coefficients of variation (CV) were determined (Table 1). The CV values for the yield-related indicators of RSA-01 were ranked as follows :leaf/stem ratio > plant height > fresh yield > dry hay yield > stem thickness > fresh/dry ratio > overwintering rate > branch number. The branch number showed the least variation, followed by the overwintering rate, whereas the leaf/stem ratio and plant height showed higher variation. The branch number, overwintering rate, leaf/stem ratio, and plant height values ranged approximately from 10 to 13, from 95.66% to 97.26%, from 0.38 to 0.45, and from 56.69 cm to 69.34 cm, respectively, indicating that variation in the leaf/stem ratio and plant height of RSA-01 were relatively high.

The CV values for the yield-related indicators of RSA-02 were ranked as follows: leaf/stem ratio > fresh yield > dry hay yield > plant height > fresh/dry ratio > stem thickness > overwintering rate > branch number. The branch number, overwintering rate, stem thickness, fresh/dry ratio, and plant height exhibited limited variation, whereas the leaf/stem ratio exhibited the highest variation. The branch number, plant height, and stem thickness values ranged approximately from 9 to 12, from 62.88 cm to 66.56 cm, and from 2.39 mm to 2.45 mm, indicating that these traits exhibited more stable performance than others.

The CV values for the yield-related indicators of RSA-03 were ranked as follows: plant height > dry hay yield > fresh yield > leaf/stem ratio > fresh/dry ratio > stem thickness > overwintering rate > branch number. Plant height exhibited the highest variation, with a CV value of 5.81%, whereas other yield-related indicators exhibited limited variations, with CV values ranging from 0.13% to 2.98%. The plant height ranged from 65.11 cm to 72.93 cm, indicating a relatively wide variation in the plant height of RSA-03. Notably, the genetic traits of other yield-related indicators were relatively stable.

To understand the effect of yield-related indicators on the dry hay yield of each hybrid strains, coefficients of correlation (*R* values) were determined (Table 2). The *R* values of dry hay yield and other indicators of RSA-01 were ranked as follows: fresh yield = plant height > stem thickness > branch number > leaf/stem ratio > fresh/dry ratio > overwintering rate. Dry hay yield was significantly positively correlated with branch number, plant height, and fresh yield at a *P* value of <0.01 and with stem thickness at a *P* value of <0.05.

The *R* values of dry hay yield and other indicators of RSA-02 were ranked as follows: stem thickness > branch number > plant height > fresh yield > overwintering rate > fresh/dry ratio > leaf/stem ratio. Dry hay yield was significantly positively correlated with plant height, stem thickness, branch number, and fresh yield at a *P* value of <0.05.

The *R* values of dry hay yield and other indicators of RSA-03 were ranked as follows: stem thickness = leaf/stem ratio > branch number > fresh yield > plant height > fresh/dry ratio > overwintering rate. Dry hay yield was significantly positively correlated with plant height, branch number, stem thickness, and fresh yield at a *P* value of <0.05 but was significantly negatively correlated with leaf/stem ratio.

### 3.2. Analysis of Nutritional Value of Hybrid Strains

**Figure 3:**
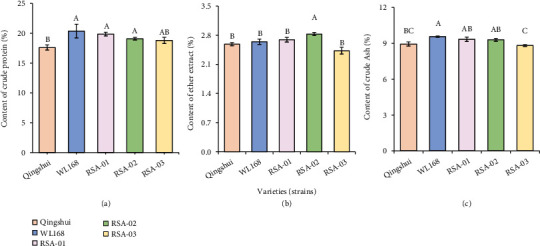
Estimated values of nutritional traits of hybrid strains: (a) crude protein, (b) ether extract, and (c) crude ash. A total of 500 g of plants was randomly selected in each plot, naturally dried, and then ground. The mean value of each nutrient-related indicator was calculated. Different capital letters indicate significant differences among varieties at *P* < 0.05.

To compare the nutritional traits of the tested varieties (strains), their CP, EE, and ash contents were measured (Figure 3). The CP contents of RSA-01 and RSA-02 were significantly higher (*P* < 0.05) than that of Qingshui by 12.78% and 8.35%, respectively, whereas the CP content of RSA-03 (18.79%) differs insignificantly from that of Qingshui. The EE contents of RSA-01 (2.69%) and RSA-03 (2.43%) differ insignificantly from that of Qingshui (2.58%, *P* ≥ 0.05), whereas the EE content of RSA-02 (2.83%) was significantly higher than that of Qingshui by 26.74% (*P* < 0.05). The ash content of the three hybrid strains (8.82%–9.33%) differs insignificantly from that of Qingshui (*P* ≥ 0.05). The mean values of the CP (19.24%), EE (2.65%), and ash (9.15%) contents of the three hybrid strains were higher than those of Qingshui by 9.26%, 2.72%, and 2.35%, respectively.

**Figure 4:**
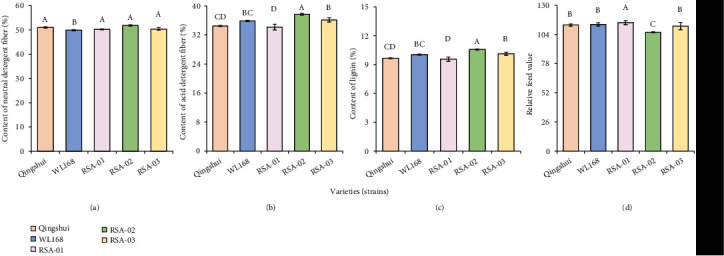
Estimated values of nutritional traits in hybrid strains: (a) neutral detergent fiber, (b) acid detergent fiber, (c) lignin, and (d) relative feed value. A total of 500 g of plants was randomly selected in each plot, naturally dried, and then ground. The mean value of each nutrient-related indicator was calculated. Different capital letters indicate significant differences among varieties at *P* < 0.05.

The comparison of NDF, ADF, ADL, and RFV of the hybrid strains and parental Qingshui is shown in Figure 4. The NDF content of the three hybrid strains (50.24%–51.85%) differs insignificantly from that of Qingshui (50.01%, *P* ≥ 0.05). Similarly, the ADF content of RSA-01 differs insignificantly from that of Qingshui; however, the ADF contents of RSA-02 and RSA-03 were significantly higher (*P* < 0.05) than that of Qingshui by 9.42% and 4.81%, respectively. The ADL contents of RSA-02 (10.57%) and RSA-03 (10.12%) were significantly higher than that of Qingshui (9.66%), whereas the ADL content of RSA-01 differs insignificantly from that of Qingshui. The RFV of RSA-02 (5.71%) was significantly lower than that of Qingshui, whereas no significant differences in RFV were observed among RSA-01, RSA-03, and Qingshui (*P* ≥ 0.05).

The CV values of nutrient-related indicators of hybrid strains are shown in Table 3. The CV values for RSA-01 were ranked as follows: ADL > ADF > EE > Ash > CP > NDF. Except for ADL and ADF, all nutrient-related indicators showed a limited variation of 0.60%–3.43%. The CV values for RSA-02 were ranked as follows: EE > Ash > CP > ADL > NDF > ADF. Except for EE, all nutrient-related indicators showed a limited variation of 0.89%–2.18%. The CV values for RSA-03 were ranked as follows: EE > ADL > NDF > ADF > CP > Ash. Except for EE, all nutrient-related indicators showed a limited variation of 1.57%–2.83%.

### 3.3. Comprehensive Evaluation of Performance-Related Indicators

**Figure 5:**
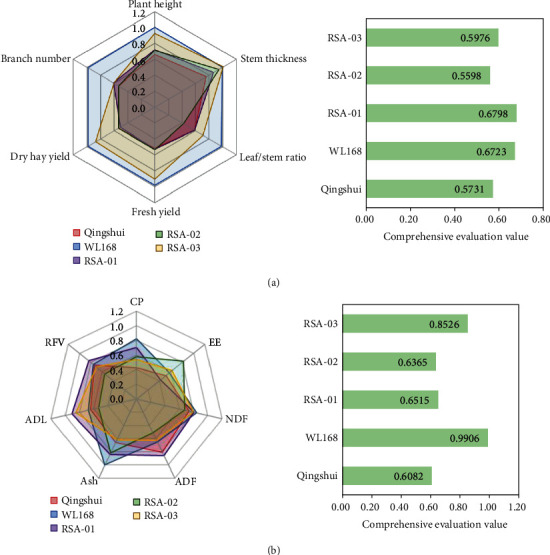
Comprehensive evaluation of production performance.

To understand the production performance of the test varieties (strains), their yield performance and nutritional value were evaluated using gray correlation degree. Because yield-related indicators were obtained in different units of measurement, the indicators were standardized to enable their direct comparison. After obtaining yield-related indices of hybrid strains, six yield-related indices were screened. The corresponding correlation coefficients were obtained via dimensionless processing using the homogenization method (Figure 5(a)). According to the weighting coefficient analysis, indicators were ranked as follows: stem thickness (0.2075) > plant height (0.1819) > fresh yield (0.1578) > dry hay yield (0.1577) > leaf/stem ratio (0.1491) > branch number (0.1479). Based on yield performance values, the test varieties (strains) were ranked as follows: WL168 > RSA − 03 > RSA − 01 > RSA − 02 > Qingshui. Thus, the parental variety Qingshui was ranked fifth, suggesting the worst performance of yield traits in this plant, whereas RSA-03 was ranked second, indicating better performance of yield traits. The plant height, stem thickness, fresh yield, and dry hay yield of RSA-03 were 12.04%, 9.25%, 24.01%, and 20.64% higher than those of parental Qingshui, respectively.

Nutrient-related performance was screened using seven indicators. The corresponding correlation coefficients were obtained via dimensionless processing using the homogenization method (Figure 5(b)). Based on the weighting factor formula, indicators were ranked as follows: Ash (0.1403) > NDF (0.1382) > lignin (0.1293) > RFV (0.1284) > ADF (0.1231) > EE (0.1056) > CP (0.1099). Based on quality traits, the test varieties (strains) were ranked as follows: RSA − 01 > WL168 > RSA − 03 > Qingshui > RSA − 02. Notably, RSA-01 was ranked first, indicating that it had the best quality performance, whereas RSA-02 was ranked fifth, indicating that it had the worst quality performance.

## 4. Discussion

### 4.1. Yield Performance of Hybrid Strains

Crop yield is affected not only by its own biological characteristics but also by external factors such as solar intensity, temperature, and fertilizer application [22, 23]. The productive performance of alfalfa is variable among different varieties but heritable between parents and offspring [16, 24–26]. Alfalfa yield is the total amount of organic matter produced and accumulated, and it can be a good indicator of production and economic performance [22, 27, 28]. In this study, 6-year-old plants were used as experimental materials as this has been determined to be the optimal age of alfalfa in semiarid areas [23, 29]. Overall, the heterosis of branch number in RSA-01, stem thickness in RSA-02, and plant height, stem thickness, branch number, and dry hay yield in RSA-03 showed better performance values than those in Qingshui, indicating that the hybrid strains inherited superior yielding characteristics of WL168. A previous preliminary study showed that RSA-03 had significant heterosis in both the aboveground biomass and stem thickness, which is consistent with the results of this study [11], indicating that RSA-03 has a prominent yield advantage. Additional studies on the advantages of different hybrid combinations in yield traits showed that, and the results varied depending on the variety used [30–32]. Thus, we speculated that yield performance indices of rhizome-rooted hybrid strains are affected by a combination of both the variety used and environmental factors.

The CV, calculated as the magnitude of genetic variation as a percentage of the mean [33], indicates the degree of variation in different variables. The CV of yield performance reflects the degree of dispersion of each trait among germplasm and is an important evaluation tool and indicator for classifying plant germplasm resources [34]. In this study, the CV value of plant height of RSA-01 was 7.48%, so the selection for forage yield is favored by the presence of large genetic variation. The CV values of overwintering rate, stem thickness, branch number, fresh/dry ratio, and plant height of RSA-02 were low, thus, essentially of easier to exploit. Except for plant height, low CV values of yield performance indices ranging from 0.10% to 2.98% were observed for RSA-03. In addition, the plant height of RSA-01 had a greater possibility of selective breeding, and the variation in RSA-03 yield-related indices was more stable. Previous studies have shown that forage yield is influenced by factors such as plant height, leaf length, fresh/dry ratio, and leaf/stem ratio, with plant height and stem thickness being positively and significantly correlated with yield [11, 22, 35, 36], which was consistent with our findings, indicating that plant height, leaf length, and fresh/dry ratio are important factors affecting alfalfa yield. Furthermore, the stability of yield performance and genetic characteristics of each hybrid selection varied because of genetic effects and climatic factors in the growing region [37].

### 4.2. Nutritional Value of Hybrid Strains

High-quality alfalfa should have three main characteristics, including high protein content, easy digestion and absorption, and good palatability [37]. CP is an essential nutrient for livestock, consisting of pure protein and nonprotein nitrogenous substances, which determines the ability of forage to meet animal protein requirements [1]. EE is the main raw material for caloric energy with an aromatic odor and is an important taste determinant in pastures [1]. Ash content represents the amount of inorganic mineral elements in the forage. ADF content affects digestibility, whereas the NDF content affects taste [29, 37]. RFV is a relatively simple index of forage quality based on ADF and NDF content and can be used to predict the intake and energy value of a forage [1, 25]. A higher RFV of alfalfa indicates better quality. In this study, the CP contents of RSA-01 and RFV of RSA-01 as well as the EE content of RSA-02 were significantly higher than those of Qingshui. Taken together, these results indicated that the nutritional value of RSA-01 was higher, whereas the palatability of RSA-02 was better. Similar to previous studies, forage productivity was increased, and its quality was improved by hybridization [27, 38]. The CV of nutrient-related indicators can reflect the stability of genetic variability traits [30, 39]. In this study, except for ADL and ADF, the CV values of nutrient-related indicators of RSA-01 were low. Similarly, except for EE, the CV values of nutrient-related indicators of RSA-02 and RSA-03 were low. Overall, the performance of hybrid strains was more stable in terms of nutrient-related indicators under variable environments, possibly because the hybrid strains were selected according to their correlation with targeted traits.

### 4.3. Comprehensive Evaluation of Gray Correlation Degree

Breeding objectives should be closely integrated with production needs [25]. Breeders should develop excellent varieties that can contribute to national economy and ecological development [10, 26]. High-quality and better-yielding alfalfa varieties have the potential to contribute to economic development. The comprehensive evaluation method can evaluate the yield performance and nutritional value of varieties (strains) more objectively and accurately [40], and its combination with practical applications in the field can ensure scientifically backed decision-making regarding trait selection. Compared with parental Qingshui, RSA-01, RSA-02, and RSA-03 showed relatively better yield performance, and RSA-01 and RSA-03 had better nutritional traits. Low-yielding Qingshui crossed with high-quality, better-yielding WL168-produced abundant hybrid progenies under the influence of genetic and environmental factors [11, 29]. Notably, crossing with WL168 improved the production performance of the naturally low-producing parental Qingshui. Rhizome-rooted alfalfa is suitable for the establishment of grazing pastures and ecological vegetation; however, we have studied the yield traits by cultivated planting, which may be considered the limitations of this study. For future studies, it is suggested to be investigated in natural or artificially grazed grasslands. Moreover, the research content such as longevity, drought, and cold resistance, as well as trampling tolerance of the hybrid strains, can be explored, comprehensively analyzing their productive performance and ecological value.

## 5. Conclusions

The evaluation of relevant yield indices in this study revealed that the tested varieties were performed in the order of WL168 > RSA − 03 > RSA − 01 > RSA − 02 > Qingshui, whereas based on nutritional values, these varieties were ranked in the order of RSA − 01 > WL168 > RSA − 03 > Qingshui > RSA − 02. Compared with the parental variety Qingshui, RSA-01, RSA-02, and RSA-03 showed better yield performance, whereas RSA-01 and RSA-03 had better quality traits. RSA-01 showed excellent advantages in terms of branch number, CP content, and RFV, whereas RSA-02 had superior stem thickness. In addition, improved plant height, stem thickness, branch number, fresh yield, dry hay yield, and CP content were observed for RSA-03, with the best comprehensive performance and suitable for wide use. The low production performance of Qingshui was improved after crossing it with WL168. Based on this study, further studies are expected to explore the longevity and stable yield performance as well as the ecological value of the rhizomatous hybrid strains from morphological, physiological, and molecular perspectives.

## Figures and Tables

**Table 1:** Coefficients of variation of yield-related indicators.

Varieties (strains)	Plant height (%)	Stem thickness (%)	Leaf/stem ratio (%)	Branch number (%)	Overwintering rate (%)	Fresh yield (%)	Dry hay yield (%)	Fresh/dry ratio (%)
Qingshui	10.17	7.30	10.40	0.10	0.78	5.82	10.94	5.40
WL168	4.25	2.83	3.61	0.10	0.32	6.34	6.86	0.66
RSA-01	7.48	4.28	7.99	0.10	1.75	7.43	4.84	3.47
RSA-02	3.27	3.04	6.76	0.10	1.69	5.42	4.38	3.23
RSA-03	5.81	1.21	2.74	0.13	1.17	2.76	2.98	1.80

**Table 2:** Coefficients of correlation between dry hay yield and yield-related indicators of hybrid strains.

Hybrid strains	Plant height	Stem thickness	Branch number	Leaf/stem ratio	Overwintering rate	Fresh yield	Fresh/dry ratio
RSA-01	1.000^∗∗^	0.997^∗^	0.996^∗^	−0.793	0.435	1.000^∗∗^	−0.582
RSA-02	0.946^∗^	0.988^∗^	0.977^∗^	−0.137	0.740	0.794^∗^	−0.830
RSA-03	0.926^∗^	1.000^∗^	0.988^∗^	−1.000^∗^	0.437	0.941^∗^	−0.781

^∗^ indicates significant correlation between dry hay yield and yield-related indicators at a *P* value of <0.05; ^∗∗^ indicates significant correlation between dry hay yield and yield-related indicators at a *P* value of <0.01.

**Table 3:** Coefficients of variation of nutrient-related indicators.

Varieties (strains)	CP (%)	EE (%)	Ash (%)	NDF (%)	ADF (%)	ADL (%)
Qingshui	2.21	10.07	3.35	0.89	0.73	3.35
WL168	5.98	1.22	1.09	0.91	0.82	1.09
RSA-01	2.62	3.43	3.42	0.60	4.27	4.98
RSA-02	1.23	3.77	2.18	0.89	1.00	1.11
RSA-03	1.67	4.69	1.57	1.94	2.80	2.83

## Data Availability

The datasets used and/or analyzed during the current study are available from the corresponding author on reasonable request.

## Supplementary Materials

Supplementary Materials Table S1: morphological traits of test varieties.
