# Supplementary material for: Cross-Breeding Improvement and Performance Analysis of Dominant Production Traits in Grazing-Type Alfalfa (Medicago sativa L.)
Source: Biomed Res Int. 2022 Nov 10;2022:1252310. doi: 10.1155/2022/1252310 (PMC9671732; doi:10.1155/2022/1252310)
Supplement: Supplementary Materials — Table S1: morphological traits of test varieties. [file 1252310.f1.docx]

**Supplementary materials**

Table. S1 Morphological traits of test varieties

| Categories | | Traits |
| --- | --- | --- |
| Parental varieties | Qingshui | Slender, stiff, horizontal, and sloping stems; lax plants; relatively short plant height and few leaf volume; and horizontal or oblique roots [11]. Its germination rate is 87.77%. |
|  | WL168 | Thick and erect stems; relatively high plant height; horizontal roots; better yield and quality performance; highly adaptable variety. Its germination rate is 95.30% [11, 17,18]. |
| Hybrid strains | RSA-01 | Erect stems, the 70°–80° angle between stem and ground; relatively high plant height and many leaf volume. Its germination rate is 86.17% [11]. |
|  | RSA-02 | Semi-horizontal stems, the 30°–69° angle between stem and ground; relatively high plant height and many leaf volume. Its germination rate is 87.26% [11]. |
|  | RSA-03 | Horizontal stems, the <30° angle between stem and ground; relatively high plant height and many leaf volume. Its germination rate is 87.11% [11]. |
